# Radiotherapy versus radiotherapy combined with temozolomide in high-risk low-grade gliomas after surgery: study protocol for a randomized controlled clinical trial

**DOI:** 10.1186/s13063-019-3741-5

**Published:** 2019-11-21

**Authors:** Jingjing Wang, Ying Wang, Yan He, Hui Guan, Ling He, Xiaoli Mu, Xingchen Peng

**Affiliations:** 1 Department of Biotherapy, Cancer Center, West China Hospital, Sichuan University, Chengdu, 610041 Sichuan China; 2 Geriatrics Center, West China Hospital, Sichuan University, Chengdu, 610041 Sichuan China

**Keywords:** Low-grade glioma, High-risk, Radiotherapy, Temozolomide

## Abstract

**Background:**

It has been reported that radiation therapy (RT) followed by procarbazine, lomustine, and vincristine (PCV) chemotherapy could improve progression-free survival (PFS) and overall survival (OS) in patients with high-risk World Health Organization (WHO) grade 2 gliomas after surgery. However, procarbazine is not available in China. In clinical practice, Chinese doctors often use radiotherapy combined with temozolomide (TMZ) to treat these patients, although large-scale prospective studies are lacking. This trial aims to confirm whether RT combined with temozolomide can improve PFS and OS in high-risk patients with low-grade gliomas (LGGs).

**Methods/design:**

This is a two-group, randomized controlled trial (RCT) enrolling patients who have LGGs (WHO grade 2) and are aged 40 years or older without regard to the extent of resection or are aged younger than 40 years old with subtotal resection or biopsy. An estimated 250 patients will be enrolled. Eligible participants will be randomly assigned to receive RT alone or RT plus TMZ chemotherapy in a 1:1 ratio. The same RT will be given to all eligible participants regardless of whether they are randomly assigned to the RT group or the chemoradiotherapy (CRT) group. While in the CRT group, patients will receive adjuvant TMZ with or without concurrent radiochemotherapy. The primary outcome of this trial is PFS, and it will be analyzed by the intention-to-treat approach. Secondary outcomes include OS, adverse events, and cognitive function.

**Discussion:**

The objective of our research is to assess the effect of radiotherapy coupled with TMZ in high-risk patients with LGGs after surgery, compared with RT alone. Different histological types and molecular subtypes will be examined, and a corresponding subgroup analysis will be conducted. Our data can provide evidence for postoperative adjuvant therapy in patients with high-risk LGGs in China.

**Trial registration:**

Chinese Clinical Trial Registry, ChiCTR1800015199. Registered on 13 March 2018.

## Background

Grade 2 glioma accounts for about 15–20% of all brain tumors in adults [1]. According to the 2016 revision of the World Health Organization (WHO) classification of tumors of the central nervous system, the major pathological types of grade 2 low-grade gliomas (LGGs) include diffuse astrocytomas (wild-type *IDH1*, mutant *IDH1*, or not otherwise specified) and oligodendrogliomas (mutant *IDH1* and 1p/19q co-deletion or not otherwise specified) [2]. The treatment modalities consist of surgery followed by observation, radiotherapy, chemotherapy, or chemoradiation [3]. Although surgery can cure a proportion of patients, some patients still have recurrence after surgery, and the LGGs can even transform into high-grade gliomas. Basing on longer survival data compared with high-grade gliomas, postoperative treatment decisions about observation versus aggressive treatments must take into account possible clinical benefits and side effects which may affect quality of life. Nowadays, postoperative adjuvant treatment strategies depend on whether the patient has high-risk factors. However, high-risk factors have been changing in recent years. Formerly, six factors were considered to be high-risk (astrocytoma, age > 40 years, Karnofsky performance score [KPS] < 70, tumor dimension > 6 cm, tumor crossing midline, preoperative neurological function deficits of moderate to severe degree). Since 2015, subtotal resection and age > 40 years are regarded as main high-risk factors for LGGs [4]. Compared with the low-risk population, patients with high-risk factors need aggressive treatments after surgery. European Organisation for Research and Treatment of Cancer (EORTC) 22845 is a prospective trial to compare early radiotherapy with deferred radiotherapy [5]. A total of 314 patients with LGGs who had undergone surgical resection or biopsy were randomized to receive either early radiation therapy (RT) or delayed RT delivered when progression took place. The results showed that immediate postoperative radiotherapy improved progression-free survival (PFS) and provided better seizure control, but there was no impact on overall survival. Taken together, the results showed that patients with high-risk WHO grade 2 glioma can benefit from postoperative adjuvant RT. In addition, adjuvant RT alone and temozolomide (TMZ) alone were compared in EORTC 22033-26033 [6]. No differences in PFS were observed between the two arms. Furthermore, the question remains: Can radiotherapy combined with chemotherapy improve clinical outcomes in high-risk patients with LGG? Radiation Therapy Oncology Group (RTOG) 9802, a prospective clinical trial, indicated improvement in both PFS and overall survival (OS) with six cycles of adjuvant procarbazine, lomustine, and vincristine (PCV) chemotherapy following radiation, when compared with RT alone. In addition, the survival distribution continued to diverge over time [7, 8]. However, procarbazine is not available in China. In clinical practice, Chinese doctors often use radiotherapy combined with TMZ to treat these patients, although large-scale prospective studies are lacking. TMZ is an alkylating agent with the chemical property to cross the blood-brain barrier. One standard treatment for high-grade glioma is to take TMZ as concurrent and adjuvant chemotherapy [9–11]. However, in LGGs, further studies are indispensable to ascertain the role of TMZ in addition to RT. RTOG 0424, a single-arm phase II study, combined concurrent and adjuvant TMZ with RT to treat patients with LGG with at least three risk factors for relapse (age ≥ 40 years, preoperative tumor diameter ≥ 6 cm, astrocytoma histology, tumor crossing the midline, or a preoperative neurological deficit of more than mild extent) [12]. The 3-year OS rate was 73.1%, which significantly exceeded not only historical controls but also hypothesis. Although RTOG 0424 unveiled that radiotherapy followed by TMZ chemotherapy for high-risk LGGs had survival benefits, it was a single-arm trial, and Chinese data is still lacking. As a result, we decided to carry out a randomized controlled trial (RCT) to confirm the advantage of TMZ chemotherapy added to RT for patients with high-risk LGGs.

## Methods/design

### Study objective

The aim of this study is to compare the efficacy and safety of RT plus TMZ with RT alone for high-risk LGGs. Beyond that, we will focus on molecular features, in addition to histologic classification, to establish a more appropriate treatment modality for certain cohorts.

### Study design

**Fig. 1:**
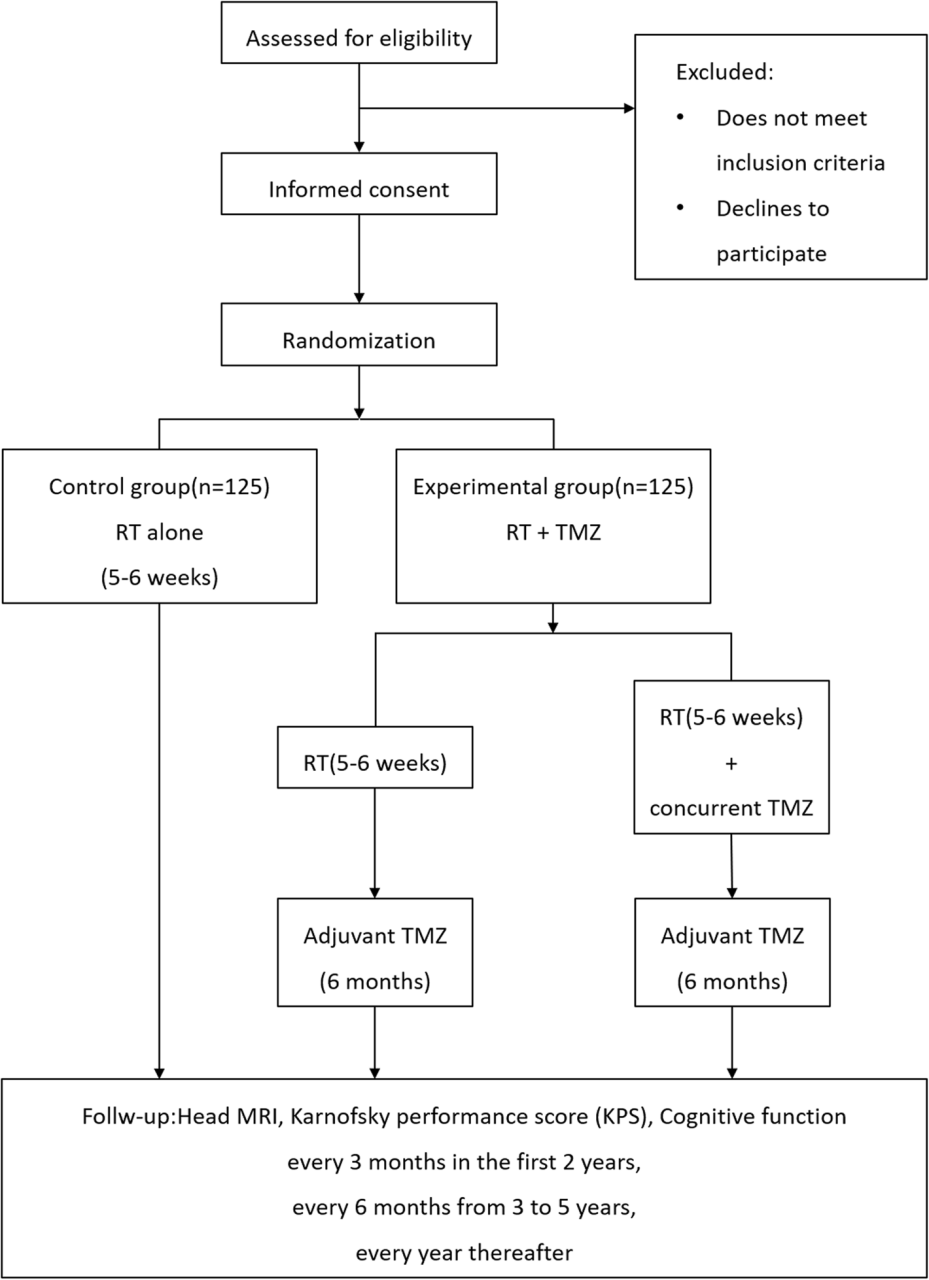
Study flowchart. *RT* radiation therapy, *TMZ* temozolomide

This is a multicenter, open-label, prospective RCT. The study has received ethical approval from the Chinese Ethics Committee of Registering Clinical Trials. The protocol is written in line with the Standard Protocol Items: Recommendations for Interventional Trials (SPIRIT) guidelines (see Additional file 1: SPIRIT checklist). Following informed consent, eligible patients will be allocated to an RT group (control group) or a chemoradiotherapy (CRT) group (experimental group). The flow diagram of the main procedures is illustrated in Fig. 1.

### Recruitment and informed consent

A total of 250 participants will be recruited from four centers in China (see Additional file 2). Registration can be done at any time after a patient is diagnosed as having a WHO grade 2 glioma. The enrollment period is expected to be completed within 5 years from the beginning of recruitment. Research staff are in charge of the screening process to make sure each participant matches the inclusion and exclusion criteria. An informed consent form (ICF) describing the detailed study procedures and illustrating the potential benefits and risks will be provided to all participants, so that they can decide whether or not to volunteer. A signed written ICF must be acquired from all patients or their legal representatives prior to their participation in this clinical trial.

### Eligibility criteria

The inclusion criteria are as follows:

1. Newly diagnosed supratentorial WHO grade 2 glioma
2. Aged 18–39 years without total resection, or aged 40–70 years with any extent of resection or biopsy
3. KPS ≥ 60
4. No more than moderate neurological symptoms or signs
5. The interval between surgery and randomization is less than 12 weeks
6. Have signed the ICF.

The exclusion criteria are as follows:

1. WHO grade 1 glioma or high-grade glioma according to WHO’s grading system
2. Patient has received prior RT to the head and neck region
3. Patient has received prior chemotherapy
4. Synchronous multiple primary malignant tumors excluding carcinoma of the cervix in situ or non-melanomatous skin cancer
5. Patient’s prior malignancy disease-free survival less than 5 years
6. Patient has active infection
7. Patient is pregnant or breast-feeding.

### Randomization

Qualified patients will be randomized in a 1:1 ratio using the method of block randomization. The blocked randomization sequence is generated by the SPSS computer software, and the block size is determined by statisticians. After the patient has registered and signed the ICF, he or she will be allocated to the RT group or the CRT group by web-based central randomization. The allocation sequence is unavailable to the researchers who are enrolling participants or assigning interventions.

### Interventions

Eligible patients will be assigned 1:1 to the experimental (CRT) group or the control (RT) group. In the experimental group, patients will be treated with RT combined with adjuvant TMZ with or without concurrent TMZ. In the control group, patients will only have RT treatment. Both groups will receive the same intensity-modulated radiation therapy (IMRT). The radiation dose is 50–54 Gy given in 25–30 fractions (1.8–2.0 Gy once daily, 5 days per week). RT treatment volumes will be defined using preoperative and postoperative T2 or fluid-attenuated inversion recovery (FLAIR) on magnetic resonance imaging (MRI). Drug dose adjustments are allowed according to blood counts and adverse reactions. Concurrent chemotherapy is to receive oral TMZ, 75 mg/m^2^ per day, during RT. Concurrent TMZ can be used for 42 days continuously if the absolute neutrophil count (ANC) is not less than 1.5 × 10^9^ cells per L, the platelet count is not less than 100 × 10^9^ cells per L, and there is a non-hematological toxicity of grade 0 or 1 (except for alopecia, nausea, and vomiting) according to version 4.03 of the National Cancer Institute Common Toxicity Criteria (NCI-CTC). In the case of ANC less than 1.5 × 10^9^ cells per L but more than 0.5 × 10^9^ cells per L, platelet count less than 100 × 10^9^ cells per L but more than 10 × 10^9^ cells per L, or a non-hematological toxicity of grade 2, concurrent TMZ treatment should suspend until recovery to toxicity of grade 0 or 1. TMZ chemotherapy will be terminated when one of the following conditions occurs: ANC < 0.5 × 10^9^ cells per L, platelet count < 10 × 10^9^ cells per L, or non-hematological toxicity of grade 3 or 4.

Patients who are assigned to adopt adjuvant chemotherapy will be treated with six cycles of TMZ, 150–200 mg/m^2^ per day for 5 consecutive days, repeated every 4 weeks. There is a 28-day break during RT and adjuvant TMZ. The first cycle dose of adjuvant chemotherapy is 150 mg/m^2^. A higher dose of 200 mg/m^2^ is recommended in the subsequent cycles if ANC is not less than 1.5 × 10^9^/L, platelet count not less than 100 × 10^9^/L, and non-hematological toxicity of grade 0 or 1 (except for alopecia, nausea, and vomiting) during the first cycle. The dose will reduce by 50 mg/m^2^ if ANC is less than 1 × 10^9^ cells per L, platelet count is less than 50 × 10^9^ cells per L, or there is non-hematological toxicity of grade 3 during any period of adjuvant chemotherapy. Patients must discontinue adjuvant TMZ if grade 4 non-hematological is recorded, the dose of TMZ has reduced to less than 100 mg/m^2^, or grade 3 non-hematological reoccurs after dose reduction.

### Outcomes

Our primary outcome is PFS, which is calculated from the date of randomization to the date of first reported disease progression or the date of death from any cause. Secondary outcomes are OS, adverse events, and cognitive function. OS is calculated from the date of randomization until death from any cause. Cognitive function will be assessed by the Mini-Mental State Examination (MMSE).

### Adverse events (AEs)

Investigators should explain to participants in detail that they are required to faithfully reflect changes in their condition during and after treatment. Researchers should pay close attention to AEs while observing the curative effect. The following information should be recorded in the case report form (CRF): symptom, occurrence time, severity, duration, treatment measures, and outcomes. Researchers should evaluate the association between AE and treatment and record them timely and truly with signature and date. The grading of AEs will be in accordance with CTC-AE version 4.03.

### Baseline and follow-up visits

After obtaining written informed consent, a baseline assessment including a physical examination, KPS, complete blood count (CBC), and serum biochemistry will be completed within 7 days before interventions. Postoperative head MRI and cognitive function test are allowed to complete within 28 days prior to treatment. During treatment, CBC and serum biochemistry (including renal and liver function, electrolytes, and lactate dehydrogenase) will be conducted weekly. The first follow-up visit (KPS, head MRI, cognitive function) will be 1 month after radiotherapy. These visits will then occur every 3 months during the first 2 years, every half year from 3 to 5 years, and at least annually thereafter. Participants will be followed up for 10 years after the end of treatment for the last patient. The schedule of assessments before, during, and after treatment is displayed in Table 1.

**Table 1:** Study schedule of enrollment, interventions, and assessments

	Study period							
	Enrollment	Allocation	Post allocation					
Time point	−4 weeks	0	Week 0–6	Week 10	Weeks 11–34	Within 2 years	3–5 years	5–10 years
Enrollment:								
Eligibility screen	X							
Informed consent	X							
Randomization	X							
Allocation		X						
Interventions:								
RT			X					
Concurrent TMZ			X					
Adjuvant TMZ				X	X			
Assessments:								
Physical examination		X				Every 3 months	Every 6 months	Annually
KPS		X		X		Every 3 months	Every 6 months	Annually
CBC		X	Weekly	X	Weekly			
Serum biochemistry		X	Weekly	X	Weekly			
Postoperative head MRI	X			X		Every 3 months	Every 6 months	Annually
Cognitive function	X			X		Every 3 months	Every 6 months	Annually
Adverse events			X	X	X	Every 3 months	Every 6 months	Annually

*Abbreviations*: *RT* radiation therapy, *TMZ* temozolomide, *KPS* Karnofsky performance score, *CBC* complete blood count, *MRI* magnetic resonance imaging

### Sample size

This RCT is designed as a superiority trial. Based on literature reports and clinical experience, the median OS of the RT group is 7.8 years and that of the CRT group (RT plus TMZ) is 13.3 years. One-sided log-rank testing with a significance level of 0.05, a test power of 80%, and a withdrawal and loss of follow-up rate of 10% is applied in this trial. A 10-year follow-up for patient events will be conducted after the end of treatment for the last patient. The sample size estimated by PASS 11 software is 125 subjects per group.

### Data collection and management

All relevant information for each subject should be recorded in the CRF and inputted in ResMan, an Internet-based electronic data capture system, timely and truly by trained research staff. The personal information of each subject is confidential. In order to promote participant retention, researchers will instruct subjects to take their medication as prescribed. Patients will also be informed of the follow-up visits by telephone in advance, and all items will be measured in strict accordance with the assessments schedule shown in Table 1. Two data entry staff are needed to input the data independently. After reviewing and confirming that the database is correct, electronic data will be conserved and backed up. As original material, the CRF is not easily changed. The researcher has to sign and date the CRF when it is necessary to modify. The locked electronic data files do not allow any changes to be made. The database will be statistically analyzed by statistical analysts as required by the statistical plan. The principal investigator has access to the final dataset, while other investigators are prohibited from entering. Except for the name-related data, the disclosure of the information to third parties is prohibited. After the completion of the trial, the responsible unit of the study has the right to publish contents related to the experiment in the form of a paper.

### Data analysis

Professional statisticians undertake statistical analysis tasks and participate in the whole process from trial design and implementation to analysis and summary. The Kaplan-Meier method will be used to estimate median PFS and OS, and a log-rank test will be used to compare differences between the two arms. Furthermore, a Cox proportional hazards analysis will be done to estimate the hazard ratio (HR) and 95% confidence interval (CI). Regarding prognostic factors, univariate and multivariate analyses including age, histology, treatment method, *IDH* mutation, 1p/19q status, and *MGMT* promoter status will be used to analyze their impact on PFS and OS. Safety analysis, mainly for AEs, will be done in the safety set (SS) population. All effectiveness analysis (PFS, OS, cognitive function) will be done on an intention-to-treat (ITT) set. The ITT analysis will be put to use to handle non-compliance and missing data.

### Data monitoring

To ensure the safety and validity of the trial, the data will be overseen by an independent Data Safety Monitoring Board (DSMB) during the study period. The board consists of clinicians and statisticians and will monitor all implementation activities including but not limited to the enrollment of each center, starting time of procedures, and drop out. All AEs and issues concerning interventions will be reported to the DSMB in line with requirements. All data entered into the database will be checked by the DSMB before being locked, and no changes will be permitted. To ensure data security, data must be backed up in time, and irrelevant personnel cannot access and modify data.

## Discussion

Optimal adjuvant management of adult low-grade gliomas (LGGs) is controversial. RTOG 9802 has shown striking survival improvements for patients with LGGs treated with adjuvant RT followed by PCV chemotherapy, and there is a significant average MMSE score increase in both arms [13]. But obviously, the incidence of AEs in the CRT group is higher than in the RT group. Thus, it is crucial to weigh the efficacy and safety of these treatments and further clarify how to combine them. Pathological molecular typing is an essential component of diagnosis and treatment of glioma at present. This clinical trial is the first large-scale prospective study to compare the effect of RT alone with RT plus TMZ involving molecular subtypes in high-risk patients with LGGs. The outcomes of this trial are expected to evaluate the predictive effects of diverse molecular markers (*IDH1*/*IDH2* mutations, 1p/19q co-deletion, *MGMT* promoter methylation status) and to find corresponding appropriate treatment patterns.

Cognitive function has aroused extensive concern in patients with brain tumors. EORTC 22033-26033, a prospective study of patients with LGGs, revealed no significant difference between the RT group and the TMZ group [14]. Therefore, it did not back the treatment of TMZ alone over RT alone. In our study, the MMSE, a widely used screening test for dementia and cognitive dysfunction and a practical approach for ranking the cognitive state [15–18], will be applied to assess cognitive function in both randomly assigned arms. It may affect the choice of individual therapeutic strategy for patients with LGGs if RT plus concomitant and adjuvant TMZ improves survival outcomes without additional cognitive function damage compared to RT alone.

### Trial status

The final protocol version is 1.0, dated 11 February 2018. Patient recruitment began on 10 April 2018 after we acquired ethical approval, and it is ongoing. We anticipate the recruitment phase to be complete by April 2023.

## Supplementary information

**Additional file 1.** SPIRIT 2013 checklist: recommended items to address in a clinical trial protocol and related documents.

**Additional file 2 Research centers.**

## Data Availability

The datasets used and/or analyzed during the current study are available from the corresponding author on reasonable request.

## Abbreviations

AE: Adverse event
ANC: Absolute neutrophil count
CBC: Complete blood count
CI: Confidence interval
CRF: Case report form
CRT: Chemoradiotherapy
DSMB: Data Safety Monitoring Board
FLAIR: Fluid-attenuated inversion recovery
HR: Hazard ratio
ICF: Informed consent form
IDH: Isocitrate dehydrogenase
IMRT: Intensity-modulated radiation therapy
ITT: Intention-to-treat
KPS: Karnofsky performance score
LGG: Low-grade glioma
MMSE: Mini-Mental State Examination
MT: Mutant
NCI-CTC: National Cancer Institute Common Toxicity Criteria
OS: Overall survival
PCV: Procarbazine, lomustine, and vincristine
PFS: Progression-free survival
RCT: Randomized controlled trial
RT: Radiation therapy, radiotherapy
SPIRIT: Standard Protocol Items: Recommendations for Interventional Trials
SS: Safety set
TMZ: Temozolomide
WT: Wild-type
