# Supplementary material for: Radiotherapy versus radiotherapy combined with temozolomide in high-risk low-grade gliomas after surgery: study protocol for a randomized controlled clinical trial
Source: Trials. 2019 Nov 21;20:641. doi: 10.1186/s13063-019-3741-5 (PMC6868800; doi:10.1186/s13063-019-3741-5)
Supplement: Supplementary file 2 — Additional file 2 Research centers. [file 13063_2019_3741_MOESM2_ESM.docx]

# Additional file 2：

| **Research setting** |
| --- |
| **West China Hospital, Sichuan University, Sichuan Province, China** |
| **The First Affiliated Hospital of Chongqing Medical University, Chongqing Municipality, China** |
| **The Affiliated Cancer Hospital, School of Medicine, UESTC, Sichuan Province, China** |
| **People's Hospital of Sichuan Province, Sichuan Province, China** |
